# Convalescent human plasma candidate reference materials protect against Crimean-Congo haemorrhagic fever virus (CCHFV) challenge in an A129 mouse model

**DOI:** 10.1016/j.virusres.2024.199409

**Published:** 2024-06-01

**Authors:** Sarah Kempster, Mark Hassall, Victoria Graham, Emma Kennedy, Stephen Findlay-Wilson, Francisco J. Salguero, Binnur Bagci, Nazif Elaldi, Murtaza Oz, Tuba Tasseten, Frank W. Charlton, John N. Barr, Juan Fontana, Chinwe Duru, Ernest Ezeajughi, Paul Matejtschuk, Ulrike Arnold, Yemisi Adedeji, Ali Mirazimi, Roger Hewson, Stuart Dowall, Neil Almond

**Affiliations:** a Science Research and Innovation, Medicines and Healthcare products Regulatory Agency, Blanche Lane, South Mimms, EN6 3QG, UK; b Virology & Pathogenesis group, UK Health Security Agency, Manor Farm Rd, Porton Down, Salisbury SP4 0JG, UK; c Sivas Cumhuriyet University, Sivas, Turkey; d School of Molecular and Cellular Biology, Faculty of Biological Sciences, University of Leeds, Woodhouse Lane, Leeds, LS2 9JT, UK; e Astbury Centre, Astbury Centre for Structural Molecular Biology, University of Leeds, Woodhouse Lane, Leeds, LS2 9JT, UK; f High Containment Microbiology and Imaging, UKHSA, 61 Colindale Avenue, London, NW9 5EQ, UK; g Current address: Supramolecular nanomaterials and interfaces laboratory (SuNMIL), École Polytechnique Fédérale de Lausanne, Switzerland; h Public Health Agency of Sweden, Solna, Sweden; i Division of Clinical Microbiology, Department of Laboratory Medicine, Karolinska Institute, ANA Futura, Campus Flemingsberg, Stockholm, Sweden; j Faculty of Infectious and Tropical Diseases, London School of Hygiene and Tropical Medicine, London WC1E 7HT, United Kingdom

**Keywords:** CCHF, CCHFV, Immunity, Infection, Mouse model, Convalescent, Vaccine

## Abstract

Crimean-Congo Haemorrhagic Fever Virus (CCHFV) is spread by infected ticks or direct contact with blood, tissues and fluids from infected patients or livestock. Infection with CCHFV causes severe haemorrhagic fever in humans which is fatal in up to 83 % of cases. CCHFV is listed as a priority pathogen by the World Health Organization (WHO) and there are currently no widely-approved vaccines. Defining a serological correlate of protection against CCHFV infection would support the development of vaccines by providing a ‘target threshold’ for pre-clinical and clinical immunogenicity studies to achieve in subjects and potentially obviate the need for *in vivo* protection studies. We therefore sought to establish titratable protection against CCHFV using pooled human convalescent plasma, in a mouse model. Convalescent plasma collected from seven individuals with a known previous CCHFV virus infection were characterised using binding antibody and neutralisation assays. All plasma recognised nucleoprotein and the Gc glycoprotein, but some had a lower Gn glycoprotein response by ELISA. Pooled plasma and two individual donations from convalescent donors were administered intraperitoneally to A129 mice 24 h prior to intradermal challenge with CCHFV (strain IbAr10200). A partial protective effect was observed with all three convalescent plasmas characterised by longer survival post-challenge and reduced clinical score. These protective responses were titratable. Further characterisation of the serological reactivities within these samples will establish their value as reference materials to support assay harmonisation and accelerate vaccine development for CCHFV.

## Highlights

• Convalescent human plasma can protect against CCHFV challenge in a mouse model.
• The protection afforded by the convalescent plasma was titratable.
• Defining the protective level of anti-CCHFV antibodies supports vaccine development.

## 1 Introduction

Crimean-Congo haemorrhagic fever virus (CCHFV) is a tick-borne viral haemorrhagic fever infection in humans causing severe disease with a reported mortality rate of 5–83 % (Shayan et al., 2015). The virus is transmitted mainly by the *Hyalomma* tick species (Bente et al., 2013). CCHFV is a member of the *Nairoviridae* family in the *Bunyavirales* order, and its genome is composed of three single-stranded negative sense RNA segments known as small (S), medium (M) and large (L). The S segment encodes the nucleoprotein and the small non-structural protein. The M segment encodes a polyprotein precursor that is processed to two structural surface glycoproteins Gn and Gc and three other domains (mucin-like domain, GP38 domain and Nsm domain), with Gn and Gc mediating receptor binding and viral entry. The L segment encodes an RNA dependent RNA polymerase (Serretiello et al., 2020). Hazara virus (HAZV) is closely related to CCHFV and within the *Nairoviridae* family but does not cause disease in humans and therefore represents a potential model for CCHFV with the advantage of working at lower containment. Hazara virus and CCHFV also have serological cross-reactivity as demonstrated by haemagglutinin-inhibition and neutralisation assays (Casals and Tignor, 1980).

CCHFV has a wide geographical distribution and has been reported as endemic in Africa, Asia, the Middle East, and South-Eastern Europe, with transmission to humans via infected ticks or through contact with blood or tissue of infected animals (Fillatre et al., 2019), (Shahhosseini et al., 2021). Early symptoms comprise of fever, headache and myalgia, similar to other febrile illnesses. In severe cases this early phase is then followed by a haemorrhagic phase (Cevik et al., 2008). It is therefore important to diagnose CCHFV as early as possible to reduce transmission and initiate patient treatment.

CCHFV is classified as a high-priority pathogen by the World Health Organization (WHO) due to a high case fatality rate and a lack of effective medical interventions or vaccines (Mehand et al., 2018). There are however vaccines in development including sub-unit vaccines including glycoprotein (Buttigieg et al., 2014) and non-structural M-segment protein (Suschak et al., 2021) that are capable of conferring protection in animal models. Only one vaccine produced in Bulgaria, from inactivated sucking mouse brain infected material, has been administered to humans (Papa et al., 2011). Therapeutic intravenous transfer of human immunoglobulin from Bulgarian vaccinated donors, to CCHFV patients, promoted recovery (Vassilenko et al., 1990). It has also been demonstrated that administration of CCHFV hyperimmunoglobulin can reduce viral loads if administered early during infection (Kubar et al., 2011).

The serological response to CCHFV is complex and it has been noted that neutralising antibodies *in vitro* do not necessarily correlate with protection *in vivo*. Vaccination of Stat129 mice with insect cell-expressed Gn or Gc ectodomains of CCHFV resulted in detectable virus neutralising activity but this did not protect these mice against CCHFV challenge (Kortekaas et al., 2015). In a suckling mouse model, it was reported that only a subset of Gc monoclonal antibodies protected mice and, moreover, that some non-neutralising Gn monoclonal antibodies also efficiently protected animals from a lethal CCHFV challenge (Bertolotti-Ciarlet et al., 2005). During infection it has also been noted that the presence of CCHFV immunity correlates with survival (Kaya et al., 2014). More recently mapping of anti-CCHFV monoclonal antibodies has revealed six distinct antigenic sites on the Gc glycoprotein. Mice administered monoclonal antibodies before or after challenge with CCHFV were protected demonstrating both the prophylactic or therapeutic use of anti-CCHFV monoclonal antibodies as therapies (Fels et al., 2021).

Given this complexity in serological responses to CCHFV it is important to understand the repertoire of antibodies produced either in response to vaccination or infection with CCHFV and identify those responses associated with protection as it would support the development of robust diagnostic clinical assays and vaccines.

One component of this process would be the development and provision of well characterised serological reference materials for CCHFV. The availability of such reference materials would help in the comparative analysis of the performance of different assays and potentially harmonise measurement, enabling comparison of CCHFV assays at different geographical locations and over time.

Herein we describe the characterisation of CCHFV convalescent plasma and particularly its capacity to protect against CCHFV challenge in a mouse model, as component steps in the development of CCHFV reference materials including candidate WHO International Standards.

## 2 Methods

### 2.1 Collection of convalescent CCHFV plasma

Patient records were searched on patient electronic chart system of Sivas Cumhuriyet University Hospital (SCUH), a large teaching hospital and one of the Turkish Ministry of Health referral hospitals for CCHF treatment (Sivas, Turkey). Individuals aged 18-year and over with a known past CCHFV infection confirmed by commercial assay RealStar® CCHFV RT-PCR Kit 1.0 (Altona Diagnostics, Hamburg, Germany) during their hospitalisation period were invited to donate plasma at the Blood Bank Unite of SCUH. The study was conducted in accordance with the declaration of Helsinki and was approved by the local Research Ethics Committee of the Ankara Numune Education and Research Hospital, Turkey (Protocol # 17–1338). A total of 7 convalescent stage of individuals were accepted to donate. Four individuals were two years post infection (past infection in 2017), and three were four years post infection (infection in 2015). The individuals were informed of the study aims and once written informed consent obtained donated a unit of blood (overall 450 mL). After donation plasma was separated by centrifugation into commercial sterile plasma bags (200 – 250 mL plasma). Donations were assigned a unique identifier (001 to 007 followed by year of infection) to establish anonymity of the donor to subsequent users of the collected materials. A plasma donation from a UK blood donor with no known previous exposure to CCHFV was used as a negative control.

After donation, plasma samples were tested for CCHFV by RT-PCR (as above) and routine blood-borne hepatitis B (HBs Ag, anti-HBs), hepatitis C (anti-HCV) and human immunodeficiency virus (HIV)1–2 viruses by commercial ELISA tests were performed. All donations tested negative except one plasma collected from a hepatitis B vaccinated female (anti-HBs positive). Anonymised plasma samples were immediately transferred to a freezer (−20 °C) overnight before storage at-80 °C until shipment. Samples were transferred at the hospital by using the standard guidelines developed by the local IBC and the USA Centers for Disease Control and Prevention (CDC) (Siegel et al., 2007) and International transport of serum samples between Turkey and the UK was performed by using regulated WHO international guidelines, (Guidance on regulations 2021).

A proportion of each individual donated plasma were aliquoted and stored at −20 °C. An equal volume of each donation was pooled and 250 µL aliquots freeze dried to produce a candidate reference material for further evaluation alongside individual donations.

The freeze drying was performed in a Telstar Lyobeta 15 dryer (Telstar Azbil, Terrassa, Spain) in 2.5 mL type I glass ampoules (Adelphi Pharmaceutical Packaging, Haywards Heath, UK). The cycle comprised of ramped freezing over 2 h to −50 °C with a subsequent 2-hour soak step, primary drying at −50 °C for 2 h, then −35 °C for 41 h at 0.1 mBar before ramping over 10 h to 25 °C and secondary drying at 25 °C, 0.1 mBar for 40 h. Ampoules were backfilled with low moisture nitrogen and stoppered *in situ*, then removed and flame sealed (Ampulmatic, Adelphi, UK). Robust freeze-dried cakes were formed. Residual moisture content was evaluated by an automated coulometric Karl Fischer titration method (Mitsubishi CA-200 AquaFast system, A1-Envirosciences Ltd, Blyth, UK) and expressed as a mass/mass weight percentage over lyophilised cake dry mass, and oxygen content measured non-invasively using infra-red laser frequency modulated spectroscopy at 760 nm (Lighthouse Instruments, Charlottesville, VA, USA) against equivalent ampoules back-filled with traceable oxygen standard gases.

### 2.2 ELISAs

96-well pates were coated overnight at 4 °C with 1 µg/mL CCHF recombinant proteins Np (REC31639), Gc (REC31730) or Gn (REC31615) (NativeAntigen, Kidlington, UK). Plates were then washed with wash buffer (PBS, 0.05 % Tween-20) and blocked with 5 % milk powder in wash buffer. After 30 min blocked plates were washed again prior to addition of a two-fold serial dilution of plasma sample. Plates were incubated at room temperature for 1 h before washing and addition of anti-human-HRP (1:5000, Merck, Dorset, UK) for 1 h at room temperature. Plates were washed again before addition of TMB substrate to each well. The reaction was stopped by addition of 2 M H_2_SO_4_ and absorbance at 450 nm was read.

Two commercially available assays were utilised to assess the levels of anti-CCHFV IgG in donated plasma. The first ELISA detected anti-CCHFV nucleoprotein (NP) antibodies, wells containing immobilised recombinant CCHFV NP were used according to manufacturer's instructions (Alpha Diagnostics, Texas, USA). The second commercially available ELISA, VectoCrimea-CHF-IgG ELISA kits (Vector-Best Laboratories, Novosibirsk, Russia), does not contain information regarding the antigen utilised in the ELISA, and was used in accordance with the manufacturer's instructions.

### 2.3 Hazara virus assays

SW13 human adrenal carcinoma cells and BSR/T7 cells were obtained from ATCC and cultured at 37 °C in Dulbecco's Modified Eagle's Medium (DMEM) supplemented with 10 % foetal bovine serum (FBS), 1 % Penicillin/Streptomycin (Pen/Strep, Lonza) (‘complete’ media herein) in a humidified incubator with 5 % CO_2_. BSR/T7 cells were selected with 1 mg/ml G418 every other passage to maintain stable expression of the T7 promoter.

Recombinant HAZV encoding GFP (rHAZV-eGFP) was generated from a cDNA-based system as described previously (Fuller et al., 2019). Briefly, BSR/T7 cells were co-transfected with the cDNA constructs pMK-RQ-S-eGFP, pMK-RQ-M, and pMK-RQ-L alongside the T7 expression plasmid pCAG-T7pol using the TransIT®-LT1 transfection reagent (Mirus) as per the manufacturer's instructions. Virus-containing supernatants were harvested 120 h post-transfection and clarified by centrifugation at 800 x *g* for 15 min. Supernatants were transferred to fresh SW13 cells for a further 120 h at 37 °C in complete media. At 120 h post-infection (hpi), virus-containing supernatant was harvested, clarified, and stored in aliquots at −80 °C. Virus titres were determined by plaque assay in SW13 cells.

For Hazara virus neutralisation assays, freeze-dried pooled plasma was reconstituted in 250 μL sterile PBS, one aliquot of each convalescent plasma was heat-inactivated at 56 °C for 60 min and a second aliquot left unheated prior to assays. A negative plasma from a UK donor with no known previous exposure to CCHFV was used as a negative control. SW-13 cells were seeded onto 96 well plates and grown to 90 % confluency overnight. Duplicate samples of plasma were initially diluted 1:50 in complete media and serially diluted by doubling dilution up to 1:800. Dilutions were incubated with 2500 plaque forming (p.f.u.) of rHAZV-eGFP (MOI 0.1) for 1 h at 37 °C and added to cells for 24 h at 37 °C. Cells were imaged 24 hpi using the IncuCyte S3 live cell imaging system (Sartorius) and infection was quantified as a change in fluorescence intensity integrated across the area of fluorescence (mean integrated green intensity; green calibration units (GCU x µm^2^/well)).

### 2.4 CCHFV neutralisation assays

Freeze-dried pooled plasma was reconstituted in 250 μL sterile PBS and serially diluted 1:2 in MEM + 0.5 % BSA from 1:25 to 1:6400. The remaining individual donations of plasma [002(2017), 004(2015), 005(2017)] were heat inactivated at 56 °C for at least 30 min and serially diluted 1:2 in MEM + 0.5 % BSA from 1:12.5 to 1:3200. Positive control anti-CCHFV antibody (NR-40,288, BEI Resources) was serially diluted 1:2 in MEM + 0.5 % BSA from 1:50 to 1:12,800.

A defined concentration of CCHFV IbAr10200 in MEM + 0.5 % BSA was added to the diluted plasma at a 1:1 ratio and mixed. To determine the number of foci forming units (FFU) resulting from infection in the absence of neutralising antibodies, a virus-only control was included in which virus was mixed in a 1:1 ratio with MEM + 0.5 % BSA only. Mixtures were incubated for 1 h at room temperature.

SW-13 cells in 24-well plates were infected with 100 μL/well of the above mixes and incubated for 1 h at 37 °C. A carboy-methyl cellulose (CMC) overlay was added and the cells were incubated for 3 days at 37 °C.

After fixing the monolayer, cells were immuno-stained and foci visualised using a CCHFV-specific antibody in a peroxidase-based enzyme immuno-assay. Visualised foci were counted and the antibody titres resulting in a 50 % reduction of foci compared to the virus only control were calculated (NT_50_).

### 2.5 *In vivo* model challenge

Studies using animals were approved by the ethical review process of the UK Health Security Agency (or its predecessors), Porton Down, UK and the Home Office, UK, via project license number P82D9CB4B. Work was performed in accordance with the Animals (Scientific Procedures) Act 1986. Humane clinical endpoints consisted of paralysis; immobility; neurological signs; or 20 % weight loss from baseline. Animals meeting any of these criteria were immediately euthanised.

Sixty-six A129 (IFNa/bR-/-) mice, aged 5–8 weeks were obtained from Marshall BioResources (UK) and randomly allocated to study groups (*n* = 6), with equal number of males and females in each group. Mice were housed in groups of 3, with water and food available *ad libitum* and environment enrichment supplied.

CCHFV (strain IbAr10200) was amplified initially in suckling mouse brain and subsequently passaged in SW-13 cells (European Collection of Cell Cultures, UK). Titre was determined by foci-forming assay in Vero E6 cells, with the lowest lethal dose (LD100) found to be 10^2^ focus-forming units per mil (ffu/mL) in a volume of 100 ml. This challenge dose has been used in over 5 independent studies, with all animals not receiving an intervention meeting humane clinical endpoints, supporting the adequacy of the challenge dose (Buttigieg et al., 2014), (Dowall et al., 2016).

0.5 mLs of the pooled convalescent plasma (neat, 1:5, 1:25 or 1:125), samples 004(2015) or 005(2017) (neat, 1:5 or 1:25) or negative sera (neat) were administered via the intraperitoneal route one day prior to challenge with 10 ffu of CCHFV (IbAr10200) via the intradermal route. Prior to challenge, 0.1 mL blood was collected and processed to sera to determine the circulating antibody concentration at time of challenge. Weight and temperature were measured daily with clinical observations assessed at least twice a day. Clinical scores were assigned a numerical value and a cumulative total determined at each timepoint [healthy (0), eyes shut or discoloured (Shayan et al., 2015), ruffled fur (Bente et al., 2013), arched (Serretiello et al., 2020), lethargic (Serretiello et al., 2020), unsteady gait (Serretiello et al., 2020), dehydrated (Serretiello et al., 2020), wasp waisted (Serretiello et al., 2020), paralysis (Mehand et al., 2018)].

At the scheduled end of the study (14 days post-challenge), or upon meeting humane endpoints, animals were culled. Blood was collected into RNAprotect tubes (Qiagen, UK) and sections of liver and spleen taken and stored at −80 °C for viral load analysis by RT-qPCR. The remainder of the tissue was placed into formalin for histological analysis.

Survival analysis was performed using a Kaplan-Meier analysis with Log rank (Mantel-Cox) test, significance was set at the 5 % level.

### 2.6 RT-qPCR analysis

Tissue samples were weighed, resuspended in 1.5 mL PBS, homogenised through a Corning Netwell plate (440 mm mesh, Merck, UK) using the barrel from a 2 mL syringe. 200 μL of tissue homogenate or blood was transferred to 600 μL RLT buffer (Qiagen, Manchester, UK) for 10 min then 600 mL 70 % isopropanol was added to each sample. Tissues were further homogenised through a QIAshredder (Qiagen, Manchester, UK) by centrifugation at 16,000 x g for 2 min and RNA extracted by KingFisher Flex automatic extraction using the BioSprint 96 One-For-All Vet Kit (Indical, Leipzig, Germany) as per manufactures instructions; RNA was eluted in 100 µL AVE buffer. Samples were analysed by RT-qPCR using the TaqMan Fast Virus 1-Step Master Mix RT-PCR kit (ThermoFisher, Loughborough, UK) with the fast cycling mode and primers/probes targeting the S segment of CCHFV (Atkinson et al., 2012). Quantification of viral load was achieved using a 10-fold serial dilution of CCHFV S segment synthetic RNA [1 × 10^6^ to 1 × 10° copies /µL] (Integrated DNA Technologies, Leuven, Belgium).

### 2.7 Histopathological analysis

Spleen and liver samples were immersed in 10 % neutral buffered formalin (NBF) for at least 7 days before being trimmed and processed to paraffin wax. 4 µM sections were cut and stained with haematoxylin and eosin (H&E), scanned using a Hamamatsu S360 scanner (Hamamatsu, Tokyo, Japan) and examined using the npdi2.view software v2.9.25 (Hamamatsu, Tokyo, Japan). A qualified pathologist assessed the presence and severity of CCHFV associated lesions in the H&E stained sections of liver and spleen from each animal using a subjective scoring system [normal (0), minimal (Shayan et al., 2015), mild (Bente et al., 2013), moderate (Serretiello et al., 2020), marked (Casals and Tignor, 1980)]. The scored parameters were: a) hepatocyte necrosis and b) presence of mixed inflammatory cell infiltrates in the liver, and c) lymphocyte apoptosis/necrosis and d) presence of macrophages in the red and white pulp in the spleen.

In addition, samples were stained using the *in-situ* hybridisation RNAScope technique to identify CCHFV RNA. Briefly, slides were pre-treated with hydrogen peroxide for 10 min (room temperature), target retrieval for 15 min (98–101 °C), and protease plus for 30 min (40 °C) (Advanced Cell Diagnostics, USA). A CCHFV-specific probe (Cat No. 479,798, Advanced Cell Diagnostics, Biotechne) was incubated with the tissues for 2 h at 40 °C. Amplification of the signal was carried out following the RNAScope protocol using the RNAScope 2.5 HD Detection Kit – Red (Advanced Cell Diagnostics). Slides were digitally scanned and evaluated by digital image analysis with the Nikon NIS-Ar software (Nikon, Praha, Czech Republic) to quantify the presence of viral RNA (percentage area positively stained).

Histopathology and in-situ hybridisation RNAScope technique were carried out in an ISO9001:2015 and GLP compliant laboratory and evaluation was performed by qualified veterinary pathologists blinded to the study groups.

### 2.8 Data collection and statistical analyses

All the data obtained from ELISA (optical density), HAZV and CCHFV Neutralisation assays (NT_50_), *in vivo* model challenge and RT-qPCR (gc/mL) and histopathological analyses and outcome data for the animals were analysed using GraphPad Prism (version 9) for Windows (GraphPad Prism Software, San Diego, CA, USA). Descriptive statistics are presented as frequencies, percentages for categorical variables and as mean +/- SEM for continuous variables. In comparing the groups, the non-parametric Kruskal-Wallis with Dunn's post-test and ANOVA with post-hoc Bonferroni were used. All tests were two-tailed and *P* < 0.05 was considered significant.

## 3 Results

**Fig. 1:**
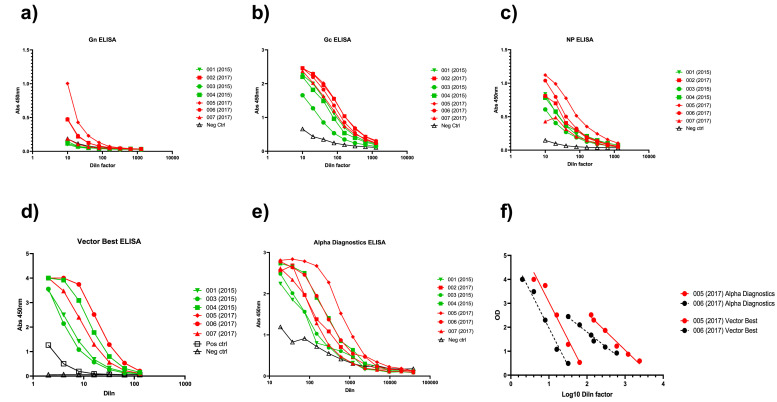
Characterisation of donations by ELISA using in-house developed ELISAs for a) Gn, b) Gc or c) Np; or commercially available assays (d and e). f) Parallel line analysis of two donation serial dilutions in commercial assays.

A total of 7 convalescent stage of individuals who have past CCHFV infection (*n* = 2, female and *n* = 5, male) were recruited and an average volume of 300 mL plasma was collected from each individual. The freeze dried material moisture content was measured by the coulometric Karl Fischer method as 0.4% w/w and the oxygen content by spectroscopy as 0.46 % which is within the recommended specifications for a WHO reference standard (Annex 2 2006). Initial serological testing demonstrated antibodies were detectable against CCHFV Np, Gn and Gc both with in-house developed ELISAs and commercially available ELISAs (Fig. 1a-e). 005(2017) had the highest binding activity against Gn and Np in ELISAs and similar binding reactivity against Gc as 002(2017) and 006(2017) by in-house ELISAs. Commercial ELISAs showed different reactivities and parallel line analysis showed that different samples, 005(2017) and 006(2017), had comparable reactivity dynamics when tested in the same assay (Fig. 1f).

**Fig. 2:**
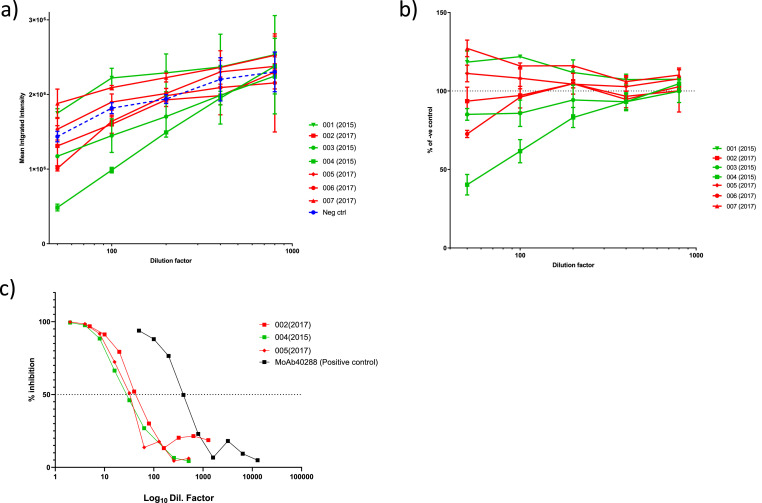
Neutralisation of HAZV by individual donations. a) Mean integrated intensity for each donation including the negative control serum and b) mean integrated intensity expressed relative to negative control serum. Bars represent means +/- SEM. c) CCHFV neutralisation curves, dotted line represents 50 % neutralisation.

Neutralisation against the related *Nairoviridae* family member, Hazara virus, showed that donation 004(2015) had the highest neutralisation capacity while 006(2017), 003(2015) and 002(2017) also had anti-Hazara virus neutralising activity, compared with a control sample (Fig. 2a and b).

CCHFV live virus neutralisation demonstrated that sample 002(2017) had the highest neutralisation capacity with an NT_50_ of 1:43. Donation 005(2017) had an NT_50_ of 1:33 and 004(2015) NT_50_ of 1:28 (Fig. 2c). The freeze-dried pooled material had the lowest neutralisation activity with an NT_50_ of 1:10.

**Fig. 3:**
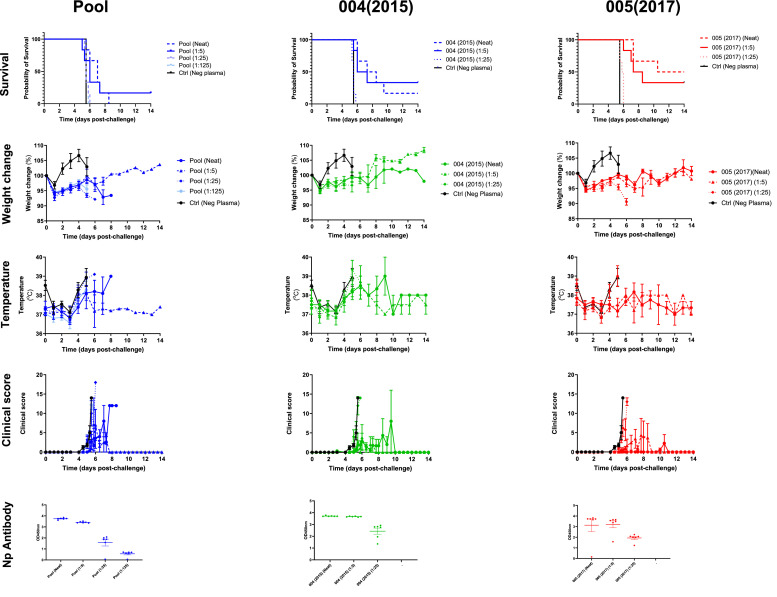
Passive protection of anti-CCHFV plasma in the A129 mouse model conferred by a pooled material (Pool) or individual donations 004(2015) or 005(2017). Parameters evaluated in the model are shown including survival, weight change (as a% change from time of challenge), temperature and clinical score. The level of anti-CCHFV antibody detectable for each individual by Np ELISA is also shown.

Three samples were taken forwards into passive immunisation studies in the mouse model: pooled plasma from the 7 plasma donations, and individual plasmas from donations 004(2015) and 005(2017). The survival of the mice up to 14 days post-challenge was assessed. Donation 005(2017) significantly increased the survival of challenged mice when administered either neat, at 1:5 or 1:25 dilution. Passive immunisation with 004(2015) also significantly increased survival after challenge when administered neat or at a 1:5 dilution but not when administered at 1:25 dilution. Pooled-freeze-dried material significantly increased survival but only when administered neat, further dilutions (1:5, 1:25 and 1:125) did not increase survival (Fig. 3).

An initial weigh decrease the day after challenge was observed in all groups, all mice then slowly gained weight with control mice gaining more weigh up to 4 days post-challenge before weight loss at day 5 and succumbing to infection. Mice that survived to 14 days continued to gain weight (Fig. 3).

An increase in temperature (1 to 2 °C) was observed in mice as clinical signs worsened. From days 4 to 6 post-challenge a temperature increase was noted for mice receiving 004(2015) neat and 1:5 dilution, this temperature increase was not observed in mice receiving 005(2017) either neat or 1:5 dilution (Fig. 3).

The level of anti-CCHFV Np antibody at time of challenge was measured by an Np ELISA and demonstrated that as a lower level of serum was administered the anti-Np reactivity also decreased.

**Fig. 4:**
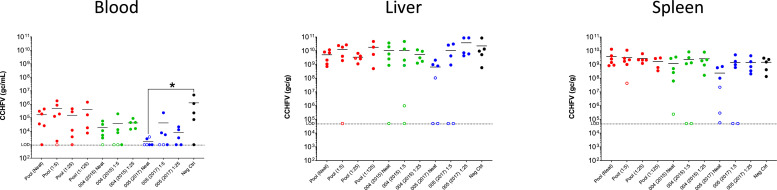
Viral RNA levels in blood, liver and spleen by rt-qPCR. Open circles represent individuals terminated at the end of the experiment (14 days) and closed circles represent individuals that met humane end points during the study. Bars represent the mean of the group and dotted lines represent the limit of detection for the assay. Asterisk denotes significantly lower level of detectable virus in mice receiving 005 (2017) neat plasma compared to the control group (*p* = 0.030).

The level of virus in blood, liver and spleen was evaluated by RT-qPCR at the time of termination. Mice that reached the end of the study (14 days) had lower levels of detectable virus in the liver and spleen. Non-parametric Kruskal-Wallis with Dunn's multiple comparison test showed a significant difference between the blood viral RNA of the control mice and mice receiving the neat preparation of serum 005(2017) (Fig. 4).

**Fig. 5:**
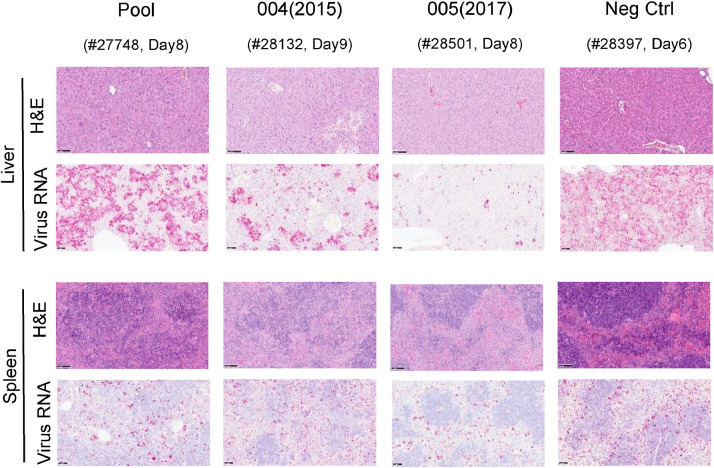
Histology images of liver and spleen from mice receiving neat plasma. Scale bars represent 100 µM. Upper panel shows H&E staining of liver and RNAScope of liver sections. Lower panel shows spleen H&E staining with RNAScope images below. Representative images from mice that met humane end points on comparable days (day of termination and animal ID noted below treatment group).

**Fig. 6:**
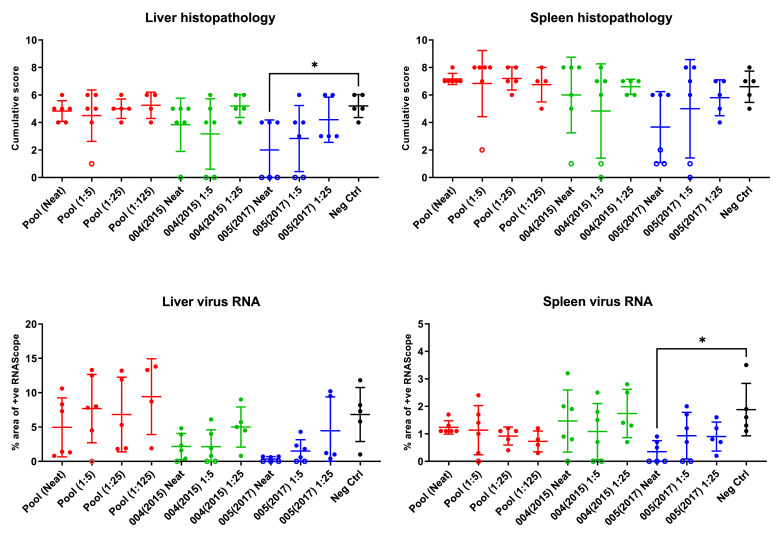
Histology scores and levels of RNAScope staining. Open circles represent individuals terminated at the end of the experiment (14 days) and closed circles represent individuals that met humane end points during the study. Bars represent the mean of the group (+/- SE). Asterisk denotes significantly lower level of detectable pathology in mice receiving 005 (2017) neat plasma compared to the control group by ANOVA with post-hoc Bonferroni (liver histopathology *p* = 0.036 and spleen viral RNA *p* = 0.019).

Upon histological analysis the main observed lesions in the liver were the presence of focal hepatocyte necrosis or multifocal hepatitis with presence of mixed inflammatory cells infiltrates, mostly on the periportal areas (Figs. 5 and 6). In the spleen, lymphocyte depletion due to cell death (apoptosis or necrosis) could be observed in the white pulp together with an increase in the number of macrophages in the white and red pulp. The presence and severity of lesions was associated to the presence of viral RNA detected by ISH RNAScope in the hepatocytes and inflammatory cells in the liver and mostly macrophages in the spleen (Figs. 5 and 6). Mice receiving the Pool material showed similar histopathological results as controls, whereas the severity of lesions and presence of viral RNA was decreased in animals receiving 004(2015) and 005(2017). Liver histopathological changes associated with CCHFV infection were significantly reduced by administration of neat 005(2017) (*p* = 0.036) and spleen viral load, evaluated by RNAScope staining quantification, was also significantly reduced in 005(2017) neat treated mice compared with controls (*p* = 0.019) (Figs. 5 and 6).

**Fig. 7:**
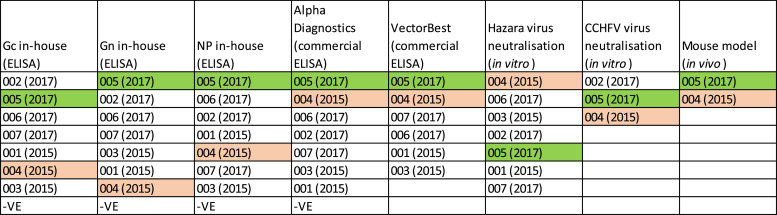
Ranking of samples by CCHFV antibody reactivity in different assays.

In summary ranking the donations performance in each assay shows that 005(2017) consistently had high levels of CCHFV reactivity, this sample did however perform less well in the Hazara virus assay (Fig. 7).

## 4 Discussion

The preparation of a candidate reference material to support clinical diagnostic assay development requires well characterised clinical sample donations with demonstratable sero-reactivity against the pathogen. Characterisation of donations from individuals with a known previous infection of CCHFV is therefore a critical part of this process. It is not only important to characterise the binding capacity of the donations but also functional sero-reactivity *in vitro* and *in vivo*, particularly if we wish to establish a potential serological correlate of protection. For CCHFV there is evidence that samples containing antibodies against Gn can protect in a pre-clinical model and that neutralisation *in vitro* does not always correlate with protection *in vivo* (Kortekaas et al., 2015), (Bertolotti-Ciarlet et al., 2005). We therefore initially characterised seven donations from individuals with confirmed CCHFV infection using ELISAs for the viral proteins Gn, Gc and Np. These antigens were selected as they represent the antigens used in commercially available anti-CCHFV diagnostic ELISAs. As a general trend the level of antibody detectable by in-house ELISAs were higher in donations from 2017 compared to those from 2015. This is not unexpected as firstly antibodies will wane over time and secondly the virus may have changed antigenically during the time frame of this study. However, the recombinant proteins used for the in-house ELISA were all HEK293 cell expressed protein sequences of the IbAr10200 isolate that was originally isolated from ticks in Nigeria in 1966 (Honig et al., 2004). CCHFV sequences obtained from ticks collected in Turkey between 2013 and 2015 were phylogenetically clustered in the Europe 1 clade that differs to this IbAr10200 isolate which is grouped with the African 1 clade (Orkun et al., 2017). CCHFV was first identified in Turkey in 2002 (Leblebicioglu et al., 2016) where Europe 1 clade continues to circulate, which may account for the incomplete protection observed in our studies, whereas challenge with a more phylogenetically-related isolate could have resulted in more potent protection *in vivo*.

The performance results of the commercial ELISAs in analysing the convalescent plasma and pooled material are more difficult to interpret, as the information on antigens included in the assays is proprietary and therefore there is the potential that different populations of antibodies within the clinical sample may be detected in different assays. Parallel line analysis demonstrated the two commercial assays have different dynamics. This could impact on the ability of a single calibrant to harmonise these assays.

HAZV belongs to the same genus as CCHFV and has been proposed as a surrogate for CCHFV studies (Dowall et al., 2012) and as such CCHFV sera was tested against HAZV infection *in vitro*. As HAZV does not infect humans it can be used at lower containment than CCHFV making it an attractive alternative. Some of the donations within this study were able to neutralise HAZV. However, the performance of a plasma sample in this assay did not correlate with its ability to neutralise live CCHFV. For example, sample 004(2015) appeared more potent than 005(2017) in HAZV neutralisation but the reverse was observed in the CCHFV live virus neutralisation assay. This suggests that whilst there are similarities between these phylogenetically related viruses, with respect to serological reactivity further work is required to harmonise these assays to ensure a firm conclusion can be drawn from these comparisons and caution should be applied when using HAZV as a surrogate for CCHFV.

Donations 005(2017) and 004(2015) were selected for passive immunisation studies in the mouse model as 005(2017) represented a ‘high’ Gn in-house ELISA reactive sample and 004(2015) a ‘low’ Gn in-house ELISA reactive sample, whilst both exhibited similar binding to Gc by in-house ELISA. The third sample for passive immunisation was a pool of all donations to represent a candidate reference material prepared from multiple donors with known previous CCHFV infection and different assay reactivities.

A titratable effect of passive immunisation against CCHFV disease was observed in the lethal mouse model as determined by survival. Control mice succumbed to disease 5 to 6 days after challenge although weight loss was only observed on the first day after challenge, after which weight gain was observed. The survival was as expected for controls and consistent for that previously observed for this CCHFV isolate and dose (Buttigieg et al., 2014) but the weight loss profile was not expected. The histopathological changes observed in the liver and spleen are similar to that described by others for this model with multifocal hepatocellular necrosis in the liver and loss of lymphocytes in the splenic white pulp (Zivcec et al., 2013). The titratable protection in the mouse model afforded by passive immunisation with 005(2017) and the differences from protection achieved by 004(2015) may be exploited for the development of clinical diagnostic assays to define a correlate of protection for CCHFV.

## 5 Conclusions

In conclusion, we have shown that convalescent plasma samples following CCHFV infection can exhibit diverse sero-reactivity profiles. Identifying which sero-reactivities are important in protection following re-exposure will inform vaccine development. Appropriate sero-reactivities also need to be present in serological reference materials if they too are going to support vaccine development and regulatory approval. A limitation of the study is that the Fc-mediated effector functions of the candidate reference materials are not characterised. Our demonstration that convalescent sera from infected individuals can confer protection in a mouse model in a titratable manner will help in the selection of component convalescent plasma that should be present in any reference materials. Further work is required to assess whether the component clinical materials described in this study can be used to produce a globally accessible reference material that supports positive developments in the vaccine and diagnostic CCHFV communities.

## CRediT authorship contribution statement

**Sarah Kempster:** Writing – review & editing, Writing – original draft, Project administration, Methodology, Investigation, Data curation, Conceptualization. **Mark Hassall:** Methodology, Conceptualization. **Victoria Graham:** Investigation. **Emma Kennedy:** Investigation. **Stephen Findlay-Wilson:** Investigation. **Francisco J. Salguero:** Writing – review & editing, Methodology, Investigation. **Binnur Bagci:** Investigation. **Nazif Elaldi:** Methodology, Investigation, Funding acquisition. **Murtaza Oz:** Investigation. **Tuba Tasseten:** Investigation. **Frank W. Charlton:** Methodology, Investigation. **John N. Barr:** Supervision, Conceptualization. **Juan Fontana:** Supervision. **Chinwe Duru:** Investigation. **Ernest Ezeajughi:** Investigation. **Paul Matejtschuk:** Methodology, Investigation. **Ulrike Arnold:** Investigation. **Yemisi Adedeji:** Methodology, Investigation. **Ali Mirazimi:** Investigation. **Roger Hewson:** Writing – review & editing, Investigation, Funding acquisition. **Stuart Dowall:** Writing – review & editing, Methodology, Investigation, Funding acquisition, Conceptualization. **Neil Almond:** Writing – review & editing, Methodology, Investigation, Funding acquisition, Conceptualization.

## Declaration of competing interest

The authors declare that they have no known competing financial interests or personal relationships that could have appeared to influence the work reported in this paper.

## Data Availability

Data will be made available on request.
